# Telemedicine as a Means to an End, Not an End in Itself

**DOI:** 10.3390/life12010122

**Published:** 2022-01-15

**Authors:** Michele Vitacca, Simonetta Scalvini

**Affiliations:** 1 Respiratory Rehabilitation Unit of the Institute of Lumezzane, Istituti Clinici Scientifici Maugeri IRCCS, 25064 Lumezzane, Italy; 2 Cardiac Rehabilitation and Continuity of Care Units of the Institute of Lumezzane, Istituti Clinici Scientifici Maugeri IRCCS, 25064 Lumezzane, Italy; simonetta.scalvini@icsmaugeri.it

**Keywords:** e-health, information communication technology, integrated care, telemonitoring

## Abstract

Telemedicine (TM)—the management of disease at a distance—has potential usefulness for patients with advanced respiratory disease. Underscoring this potential is the dramatic expansion of its applications in clinical medicine. However, since clinical studies testing this intervention often provide heterogeneous results, its role in the medical management of respiratory disorders remains inconclusive. A major problem in establishing TM’s effectiveness is that it is not a single intervention; rather, it includes a number of divergent diagnostic and therapeutic modalities—and each must be tested separately. Reflecting the discord between the need for further documentation of its approaches and effectiveness and its rapid utilization without this needed information, a major challenge is the lack of international guidelines for its integration, regulation, operational plans, and guidance for professionals. Tailored TM, with increased flexibility to address differing healthcare contexts, has the potential to improve access to and quality of services while reducing costs and direct input by health professionals. We should view TM as a tool to aid healthcare professionals in managing their patients with respiratory diseases rather than as a stand-alone substitute to traditional medical care. As such, TM is a means rather than an end.

## 1. Background

The increasing number of individuals with chronic disease poses a dramatic challenge to the sustainability of current systems providing healthcare [1]. Aggravating this problem, the increase in life expectancy in the population requires a shift from an acute illness to a chronic disease model, undoubtedly requiring enhanced effectiveness, integration, and cooperation of medical institutions [2]. To this end, improving on knowledge, sharing, and collective strategies is necessary to promote good health and quality of life for individuals with chronic diseases [3]. European economies are finally coming to realize that investing in the health of older people can contribute to the development of future generations [4]. Among chronic respiratory diseases, hospitalization for COPD ranks near the top, ranging from 40 to 60% of the total disease costs [5]. Furthermore, hospitalization mortality is high, as is the readmission rate in the year following the index hospitalization for an exacerbation [5]. Besides the morbidity and mortality risk from the disease itself, frequent exacerbations, systemic effects, and comorbidities contribute substantially to the overall burden [5]. As the average age of respiratory patients increases and in-hospital stays are shortened, the burden of home care for those sent home from the hospital with higher levels of disability [6], requirements for home mechanical ventilation [7], problems resulting from increased social frailty [6,8], and requirements needed to identify and treat relapses [9] also increase. Since health and social care systems are often disconnected, integrated approaches, including enhanced continuity of care will be necessary to optimize outcomes [10]. To meet these challenges, digital health technology (DHT) [11] in the form of telemedicine (TM) may be of benefit. 

## 2. Key Question

The main problem to be discussed in the present commentary is whether the opportunities and risks, good practices, and limitations of DHT and TM can represent an instrument to achieve better results in terms of the management of respiratory diseases or whether it can be the ultimate end of our activity to replace the conventional modality to care for patients.

## 3. Definitions

For this discussion, DHT includes digital medicine (telemedicine, telecare, wearable sensors for remote monitoring, smartphone apps, and virtual augmented reality), artificial intelligence (AI), and robotics systems and devices [12,13]. TM refers to managing a patient’s disease at a distance by incorporating technology and typically involving health professionals with different dedicated and integrated skills and tasks [12,14]. Different TM programs are available, including telenursing, telediagnosis (polysomnography, oxygen saturation via pulse oximetry, spirometry), tele-titration of mechanical ventilators, telemonitoring (to measure adherence, residual apnea–hypopnea index, leaks, and oxygenation, wearable devices to monitor different biological signs), tele-education and telerehabilitation [12].

## 4. Opportunities

The potential benefits of the extensive use of TM include a reduction in care inequity, increased personalization of services, improved health outcomes, better integration of health and social systems, increased access to healthcare, health promotion and prevention, enhanced education and empowerment, efficiency, improved adherence to treatments, and research and technological innovation [13,15]. In addition, reduced anxiety and travel burden associated with TM-assisted home therapy is a remarkable service for respiratory patients [13,15]. Further expected opportunities are primary care inclusion, reinforcement of hospital networks, investment in digital health and digital knowledge, and serious political commitment for normative and regulatory frameworks [13].

## 5. Challenges 

Concerning using TM in the integrated care of the respiratory patient, introducing new technologies or innovative services into care delivery presents obvious challenges. Challenges to the large-scale implementation of TM include a lack of international guidelines for its integration, regulation, and operational plan, insufficient guidance for professionals, and inadequate integration of information to improve epidemiological surveillance [13]. Additionally, overcoming human inertia to the wave of technological innovation [16], adequate healthcare financing accomplished through a public–private partnership [16], investment in human capital as in information and communications technology solutions [17], and the combination of health and social care services [18,19] would be welcome. In the respiratory field, there is the availability of health apps for prevention, disease management, diagnosis, treatment and care planning, tele-coaching for a healthy lifestyle, monitoring oxygenation, peak expiratory flow, symptoms, and drug adherence [20]. Additionally, TM programs, such as tele-spirometry, telemonitoring of biological signals, tele-visits, videoconferencing, and other signal transmissions, such as mechanical ventilator tracing memory, are available [15]. However, as is the case for TM in general, while these technologies are promising, to date no clear and standardized guidelines and/or recommendations are available for their routine use. Using this technology should be safe, effective, and flexible under different conditions and with different patients.

## 6. Barriers

Major barriers to TM implementation include the lack of awareness of e-health solutions and interoperability, limited available cost-effectiveness studies, insufficient legal direction, questionable or poor cost-effectiveness, risk of impairing the relationship between the patient and healthcare provider, insufficient reimbursement, and high up-front costs in terms of implementation [13,21,22]. However, integrating TM into existing healthcare, including its acceptance and utilization by healthcare teams, also presents a barrier to overcome. Interestingly, technology as such is not considered as significant an obstacle as human resistance (both laypeople and clinicians) to innovation [17]. Managing change has always been a complex endeavor; it involves adaptation to uncertain and changing situations, including the planning and implementation of available resources to meet challenges [23,24].

## 7. Published Results on Effectiveness

TM interventions have been extensively studied in COPD, chronic respiratory failure, asthma, and interstitial lung diseases. Given the different approaches to TM, the range of respiratory diseases, and different research designs, firm conclusions are problematic. According to research on the EMBASE, PubMed, and Scopus databases, reviews and meta-analyses published between 2011 and 2021 in English were considered. Table 1 and Table 2 summarize the main results on the effectiveness of TM and tele-rehabilitation in lung diseases. However, in general, positive results are demonstrated in emergency room admissions, exacerbation-related readmissions, early detection of acute exacerbation of COPD, and health-related outcomes [25,26,27,28,29,30,31,32,33,34,35,36]. Additionally, a review of tele-rehabilitation papers showed similar, favorable outcomes compared to standard, center-based pulmonary rehabilitation in functional exercise capacity, health related quality of life, and dyspnea [37,38,39,40,41]. Both interventions were safe and tele-rehabilitation patients were more likely to complete the program. Tele-rehabilitation, particularly useful in areas with poor access to center-based programs, has the capacity to promote adherence [42]. Enthusiasm over positive outcomes from TM and tele-rehabilitation compared to standard home care must be tempered by the fact that the selection of patients most suited to virtual interventions has yet to be clearly identified [15]. Another criticism is related to the definition and content of standard care to which TM is compared, as standard care can differ between countries [43]. One would expect less robust results when TM is utilized in those countries where a robust and extensive home care package already exists [15]. Moreover, different modalities of tele-monitoring and e-health platforms (from asynchronous systems to non-immediate analytical or decision-making structure to complete and constant analytical support 24 h a day, 7 days a week) may also cause differences in the final results [15].

## 8. Perspectives

With the impact of widespread infection with SARS-CoV-2 (COVID-19) beginning in 2019, standard health programs became almost unavailable. By necessity, the inertia imposed by conventional wisdom in providing standard but potentially hazardous, face-to-face medical care had to be overcome, resulting in the widespread use of TM in medical care, including in those with respiratory diseases [44,45]. In general, this led to wider acceptance of TM as a desired and beneficial therapeutic modality, leading to changes in healthcare organizations incorporating it into their systems [46].

From an international perspective, further implementation of TM depends on political buy-in and leadership from healthcare agencies to increase and disseminate digital solutions and guidelines to enhance operability, safety, collaboration, effectiveness, and infrastructure, supported with public and private capital. Its modus operandi is to align digital innovation with public health system goals [14,20,30]. Supporting these initiatives, research will be necessary to explore digital health interventions’ impact, efficacy, and cost-effectiveness—always considering the solid need for a Health Technology Assessment (HTA) [11].

## 9. Conclusions

TM use has already increasing in clinical situations where direct, one-on-one care is not feasible, such as in remote areas or when it might present an undue risk to the patient, family, or caregiver, such as during a pandemic. Additionally, it is proving useful in offering a care option that may increase patient satisfaction (and increased adherence to therapy) or even match the outcomes of standard therapy, such as tele-rehabilitation for COPD patients. Despite this emerging evidence, systematic reviews of this intervention often show inconsistent outcomes and clinical studies are often heterogeneous, making firm conclusions on outcome effectiveness and cost-effectiveness difficult to draw. For these reasons, to date, TM may not quite be the ultimate solution that was promised. Future TM research will need to better characterize the target population for each modality, demonstrate reasonable cost-effectiveness, address potential privacy, security, and legal issues, and outline clinician responsibilities. Tailored TM programs with high flexibility for different healthcare contexts would improve access and quality, reducing costs and improving health personnel’s daily work. Over time, TM will likely be considered one of several approaches to better manage respiratory patients’ disease and comorbidities. As such, it will not replace standard medical care but will be one component of a healthcare package; it will be a means to an end rather than an end in itself—to enhance accessibility, satisfaction, uptake, and clinical outcomes in individuals with chronic respiratory diseases. 

## Figures and Tables

**Table 1:** Effectiveness of telemedicine in lung diseases (reviews and meta-analyses).

Disease(s)	Outcomes	References
COPD	Reduction in emergency room visits Exacerbation-related readmissions (if TM more than 6 months) Exacerbation-related hospital days Quality of life: St. George’s Respiratory Questionnaire (SGRQ) score Early detection of acute exacerbations of COPD Health-related outcomes	[25,26,27,28]
COPD	Mortality, all-cause readmissions, rate of exacerbation-related readmissions, all-cause hospital days, time to first hospital readmission, anxiety, depression, exercise capacity	[25]
COPD/asthma	Self-management (SM) SM supported by telehealth produces reduction in healthcare utilization	[25,29,30,31]
COPD	Oxygen saturation measurements utility	[32]
Asthma	Symptoms and total dose of oral prednisolone	[33]
Respiratory diseases	Utility of noninvasive portable Digital Technologies	[34]
COPD	Use of Forced expiratory volume assessed daily, resting respiratory rate, respiratory sounds, end-tidal carbon dioxide level	[28]
COPD/asthma	Adherence to therapy	[35]
Chronic Respiratory Failure	Reduction in emergency room visits, exacerbation-related readmissions, GP urgent calls, and hospitalizations	[36]

**Table 2:** Effectiveness of tele-rehabilitation in lung diseases (reviews and meta-analyses).

Disease(s)	Outcomes	References
COPD	Health-related quality of life, exercise capacity at six and 12 months	[37]
COPD interstitial lung disease bronchiectasis	Exercise capacity (6 min walk test), St George’s Respiratory Questionnaire (SGRQ), breathlessness on the Chronic Respiratory Questionnaire (CRQ) dyspnea domain score	[38]
Cardiopulmonary diseases	Exercise capacity (6 min walk test), peak oxygen consumption, quality of life	[39]
Respiratory diseases	Safety Feasibility Reduced face-to-face rehabilitation therapy	[38,40]
Respiratory diseases	Assessment of sit-to-stand tests, Timed Up and Go step test, 6 min walk test (not for patients at risk of desaturation)	[41]

## Data Availability

Not applicable.
